# Dietary Supplementation of Olive Mill Waste Water Polyphenols in Rabbits: Evaluation of the Potential Effects on Hepatic Apoptosis, Inflammation and Metabolism through RT-qPCR Approach

**DOI:** 10.3390/ani11102932

**Published:** 2021-10-11

**Authors:** Katia Cappelli, Flavia Ferlisi, Samanta Mecocci, Margherita Maranesi, Massimo Trabalza-Marinucci, Massimo Zerani, Alessandro Dal Bosco, Gabriele Acuti

**Affiliations:** 1 Department of Veterinary Medicine, University of Perugia, Via San Costanzo, 4, 06126 Perugia, Italy; katia.cappelli@unipg.it (K.C.); flavia.ferlisi@studenti.unipg.it (F.F.); samanta.mecocci@studenti.unipg.it (S.M.); massimo.zerani@unipg.it (M.Z.); gabriele.acuti@unipg.it (G.A.); 2 Department of Agricultural, Food and Environmental Science, University of Perugia, Borgo XX Giugno, 74, 06100 Perugia, Italy; alessandro.dalbosco@unipg.it

**Keywords:** polyphenols, olive mill waste waters, liver, rabbit, RT-qPCR, gene expression

## Abstract

**Simple Summary:**

The wastes obtained from olive oil processing have a negative impact on the environment, but are rich in bioactive molecules such as phenolic compounds. These compounds have recently been used to manufacture nutritional supplements to improve animal health and welfare, productive performances, and to produce functional foods of animal origin (meat, milk, cheese). Polyphenols have antioxidant, antimicrobial and anti-inflammatory properties that modulate apoptotic pathways, cytokines, genes and protein expressions in various cellular systems. The liver is the main metabolic organ and several papers have demonstrated that it is a target organ of polyphenol molecules. The aim of this study was to highlight the effects on gene expression of inflammatory, metabolic and apoptotic effectors in the livers of rabbits fed with a polyphenolic concentrate obtained from olive mill waste waters (a residue of the extraction process of extra virgin olive oil). Quantitative Real-Time PCR results showed the down-regulation of *SIRT1*, *TNFA*, *AGER*, *BAX* and *PPARA* gene expressions in the POL group compared to the CTR group. These results show for the first time that using olive mill waste waters can prevent the harmful effects of oxidative stress in the cellular systems of food-producing animals such as rabbits.

**Abstract:**

Agro-industrial processing for the production of food or non-food products generates a wide range of by-products and residues rich in bioactive compounds including polyphenols. The concentration of these by-products is sometimes higher than in the original raw material as in the case of olive mill waste water (OMWW), one of the main by-products of olive oil extraction. Polyphenols are secondary plant metabolites that regulate the expression of specific inflammatory genes, transcriptional factors and pro/anti-apoptotic molecules, thus modulating the signaling pathways essential for cell health and homeostasis. The liver plays a key role in regulating homeostasis by responding to dietary changes in order to maintain nutritional and physiological states. In this study a nutrigenomic approach was adopted, which focuses on the effects of diet–health–gene interactions and the modulation of cellular processes, in order to evaluate the expression of the genes (*AGER*, *BAX*, *COX2*, *IL1B*, *PPARA*, *PPARG*, *SIRT1*, *TNFA*) involved in these interactions in the livers of rabbits fed with a diet supplemented with OMWW (POL) or without supplements (control, CTR). The RT-qPCR analysis showed the down-regulation of *SIRT1*, *TNFA*, *AGER*, *BAX* and *PPARA* transcripts in the POL group compared to the CTR group. These results show that OMWW dietary supplementation prevents cell death and tissue deterioration in rabbits.

## 1. Introduction

The significant reduction in environmental impact achieved by recycling agricultural waste and by-products has led to the publication of thousands of studies in the third millennium’s first two decades [1]. In this context, the circular economy (https://www.europarl.europa.eu/news/en/headlines/economy/20151201STO05603/circular-economy-definition-importanceand-benefits, accessed on 18 May 2021) is particularly important for the agro-food sector as it aims to apply new techniques to recover valuable bioactive compounds from agri-food by-products to be used in animal nutrition [2,3,4,5]. Within the agri-food chain, the extra virgin olive oil production industry generates a wide variety of waste residues which have significant environmental impacts, such as olive vegetation waters, olive pomace and olive leaves [1,6]. Moreover, olive oil waste may negatively affect soil microbial populations, aqueous ecosystems and air medium due to its high phytotoxicity [7]. However, the large number of bioactive molecules, especially polyphenols, found in olive oil by-products can be used to make nutritional supplements with the aim of improving animal reproductive and productive performance, health status and welfare, in order to obtain animal-derived functional foods for human consumption [8,9,10]. Indeed, in recent years efforts have been made to improve animal welfare by developing alternative dietary strategies based on plant-derived metabolites instead of feed additives or chemical products [11]. Rabbits efficiently convert the proteins contained in cellulose-rich plants into food containing high-value animal protein, and these are therefore a simple and sustainable protein source to produce in rural communities and economically less-developed countries [12,13]. Due to the current societal focus on sustainability and avoidance of food-feed competition, nutrient-rich functional foods that are high in polyphenols such as rabbit meat have recently attracted great interest [13]. Moreover rabbits are more suitable than other experimental animals (e.g., rodents) for studying obesity and metabolic abnormalities such as dyslipidemia, atherosclerosis, metabolic syndrome and insulin resistance, and liver dysfunctions, since their lipid profile and metabolism are similar to those of humans [14,15,16,17] and they are particularly susceptible to oxidative stress [14] which affects this organ [15]. The liver plays an important role in many metabolic processes and in rabbits it is essential for polyunsaturated fatty acid synthesis, such as that of arachidonic acid [16,17]. Hydroxy-tyrosol, a polyphenol found in olive oil by-products, has been shown to possess anti-inflammatory properties in-vitro as it inhibits the expression of the two inducible pro-inflammatory genes Nitric Oxide Synthase (NOS) and Cyclooxygenase-2 (COX2), and it has proven to have anticancer properties both in epidemiological and in-vitro studies [18].

Moreover, polyphenols regulate inflammation by modulating the expression of cytokines, such as interleukin 1β (IL1B), IL6, IL8, COX2 and the tumor necrosis factor alpha (TNFα) enzyme [19]. Many papers [20,21,22,23] have shown the pro-apoptotic and anti-apoptotic properties of these compounds.

It has recently been reported that hydroxy-tyrosol upregulated the expression of SIRT1 (a protein involved in various-cellular mechanisms such as cell reprogramming [24], DNA repair [25] apoptosis [26], glucose and lipid metabolism [27] and redox homeostasis [28]) in TNFA-induced vascular adventitial fibroblasts, which induced an anti-inflammatory response [29].

Oleuropein, another antioxidant compound of olive oil by-products, prevented LPS-induced inflammatory response in mice livers, by reducing the expression of the proinflammatory TNFA, IL1B, and IL6 cytokines [30].

Moreover, oleuropein reduced total cholesterol and triglyceride serum levels and protected the rabbits against oxidative-induced myocardial injuries [31].

These effects may be achieved through the activation of the three-peroxisome proliferator-activated receptors (PPARs alpha, delta and gamma), a nuclear receptor subfamily, which are involved in various processes such as steroidogenesis, angiogenesis, tissue remodeling, cell cycle and apoptosis [32]. PPARα is considered to be a cellular “lipostat” which regulates lipid levels and the transcriptional activity of several PPARα target genes, which restore physiological lipid concentrations [33].

Another polyphenol found in olive oil by products, resveratrol, has antioxidative and anti-inflammatory properties which can reduce oxidative stress on the liver by downregulating the receptor for advanced glycation end products (RAGE or AGER) that belongs to the immunoglobulin super family [34]. When its ligands bind to AGER, they induce intracellular oxidative stress and subsequently activate the transcription factor nuclear factor-κB (NF-κB) [35]. During one of our recent studies, evidence was found that dietary polyphenols modulate inflammatory and apoptotic activities in rabbit ovaries, thus suggesting a functional involvement of these dietary compounds in mammalian reproduction [36].

For the reasons outlined, the aim of this study was to highlight the possible effects of inflammatory, metabolic, and apoptotic effectors on gene expression in the livers of rabbits fed diets supplemented with polyphenolic concentrate obtained from olive mill waste waters (OMWW). A new strategy may be to supplement the animals’ diets with olive oil by-products recycled in an eco-friendly and sustainable way with the aim of improving animal welfare and reducing their environmental impact. 

## 2. Materials and Methods

### 2.1. Animals and Diets

The study was conducted in the “Bird-Rabbit Experimental Section” of the Department of Agricultural, Food and Environmental Sciences of the University of Perugia (Vestricciano, Perugia). Twenty-four New Zealand rabbits were weaned at 30 days of age and randomly assigned to two homogeneous dietary groups for live weight (average live weight: 652.34 ± 65.45 g) and sex. The animals were housed individually under controlled lighting conditions (16 h light/8 h darkness), temperature (18 to 23 °C) and humidity (60 ± 5%). The rabbits were either fed a commercial pelleted concentrate (control group, CTR n = 12) or the same diet supplemented with a liquid OMWW product to reach a total concentration of 282.4 mg/kg of phenolic compounds (POL group, n = 12). Experimental feeds were sampled three times (at the beginning, in the middle and at the end of the trial) following the Commission Regulation (EC) No 691/2013 and analyzed according to AOAC methods (2000). The ingredients and proximate composition of the commercial pelleted feed and the polyphenol contents in the feeds have previously been reported in Maranesi et al. [36]. The vaccination plan was developed in accordance with diagnostic tests and vaccination administration protocols established for terrestrial animals (OIE, 2012). No clinical symptoms or illnesses were observed in the rabbits throughout the whole experiment and no mortality was recorded. Fresh water was provided. The rabbits were slaughtered at 90 days of age (average live weight: CTR 2316.25 ± 214.84 g, POL 2302.53 ± 119.44 g) in a slaughterhouse according to animal welfare codes of practice and standard commercial procedures.

### 2.2. Tissue Collection and RNA Extraction

At slaughtering liver samples were collected from 24 randomly selected subjects, 12 from each experimental group, POL and CTRL. The tissues were snap frozen and lysed with pestle/mortar in liquid nitrogen. The tissue powder was then homogenized in TriZol reagent (Thermofisher Scientific, Waltham, MA, USA). Total RNA was extracted from approximately25 mg of liver homogenate using an Aurum™ Total RNA Fatty and Fibrous Tissue Kit (Bio-Rad, Hercules, CA, USA), in accordance with manufacturer specifications. RNA extraction was assessed using a Nanodrop 2000 Spectrophotometer (Thermo Fisher Scientific, Waltham, MA, USA).

### 2.3. Real Time RT-qPCR

The same amount of RNA for each sample (1 μg) was reverse-transcribed into cDNA, using the SuperScript^®^ VILO IV™ Master Mix (Thermo Fisher Scientific, Waltham, MA, USA) and following the manufacturer’s guidelines. The gene expression of specific target genes was evaluated: advanced glycosylation end-product specific receptor (*AGER*), BCL2 associated X apoptosis regulator (*BAX*), cyclooxygenase2 (*COX2*), interleukin 1 beta (*IL1B*), peroxisome proliferator activated receptor alpha (*PPARA*), peroxisome proliferator activated receptor gamma (*PPARG*), sirtuin 1 (*SIRT1*), and tumor necrosis factor-α (*TNFA*). Ribosomal RNA 18 (*RN18S*) and actin beta (*ACTB*) were used as reference genes for real time quantitative PCR (RT-qPCR), comparing the expression levels of the two groups: control (CTR) and polyphenolic byproduct-supplemented (POL). Primers were designed in accordance with the sequences available on the Primer-BLAST online design platform (https://www.ncbi.nlm.nih.gov/tools/primerblast/, accessed on 31 July 2021) and primer pairs were placed in different exons or at exon-exon junctions in order to avoid biases due to genomic DNA amplification. Specific primer pairs for the reference genome were verified in-silico using In-Silico PCR software (https://genome.ucsc.edu/cgi-bin/hgPcr, accessed on 31 July 2021) to confirm their specificity for targeting (Table 1).

A preliminary RT-qPCR reaction efficiency test was performed for each primer pair. Moreover, in order to verify the amplification of a-specific products or primer dimer artefacts, the melt curves were examined and a single well-defined peak was observed in the negative first derivative plot. The reference genes, *ACTB* and *RN18S*, were tested for their stability under the two biological conditions.

RT-qPCR reaction was performed with 5 μL of cDNA 1:10 diluted and SsoFast™ Eva-Green^®^ Supermix (BioRad, Hercules, CA, USA). Amplification was carried out with a CFX96™ Real-Time PCR detection system (BioRad, Hercules, CA, USA) under the following conditions: 95 °C for 1 min, then 40 Cycles of 95 °C for 10 s followed by 60 °C for 20 s. Fluorescence was measured at the end of the second step and the melting curve was determined at the end of the reaction by increasing the temperature by 0.5 °C every 0.05 s until it reached 95 °C.

### 2.4. Statistical Analysis

A normalization step was performed according to the expression levels of the two reference genes *18S* and *ACTB*, after assessing their stability under the two different experimental conditions, using the Norm algorithm included in Bio-Rad CFX Maestro software (ver. 4.1 BioRad, Hercules, CA, USA). Relative normalized expression was assessed using the 2^−ΔΔCT^ statistical method [37]. After normalization on the reference gene (ΔCT) set of both the supplemented and control samples, the difference between the two ΔCT values was calculated (∆∆CT). The data were analyzed using Bio-Rad CFX Maestro software (ver. 4.1 BioRad, Hercules, CA, USA) and were compared using the Student’s *t*-test. Differences were considered significant at *p* < 0.05. The equality of variances was checked using Levene’s test.

## 3. Results

### Real Time RT-qPCR

For all reactions, the range of amplification efficiency was 88–115% and the variation of the standard curve for correlation coefficients (R^2^) was 0.98–0.99. There were no primer dimer formations and only one melting peak was revealed by the melt curve analysis which confirmed the specificity of the amplification. The average M value for the reference genes was approximately 0.242 and 0.311 for *RN18S* and *ACTB*, respectively, well below the usual threshold (M-value < 1.5), confirming the appropriate choice of reference genes for this type of tissue and biological conditions.

**Figure 1:**
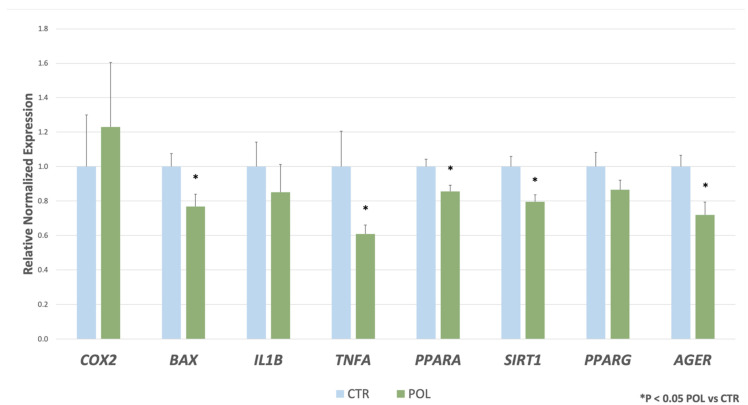
Gene expression levels of *COX2*, *BAX*, *IL1B*, *TNFA*, *PPARA*, *SIRT1*, *PPARG* and *AGER* in hepatic tissue of rabbits fed with a commercial pellet alone (CTR, blue bars) or supplemented with an olive mill waste water (OMWW) product to reach a total concentration of 282.4 mg/kg of phenolic compounds (POL, orange bars), using a 1/10-log scale. Data represent the mean ± SD. Student’s *t*-test: * *p* < 0.05, POL vs. CTR (*BAX* = 0.036, *TNFA* = 0.021, *PPARA* = 0.017, *SIRT1* = 0.007, *AGER* = 0.014).

The expression levels of *SIRT1*, *TNFA*, *AGER*, *BAX,* and *PPARA* were lower (*p* < 0.05) in POL group compared to CTR one (Figure 1). No differences between CTR and POL groups were evidenced for *COX2*, *IL1B*, and *PPARG* gene expression (Figure 1).

## 4. Discussion

This study demonstrated the potential effects of dietary polyphenols on inflammatory and apoptotic pathways in rabbit livers.

Over the last few decades these bioactive compounds have been used as feed supplements to enhance animal performance, health and welfare, and to manufacture functional foods of animal origin [3,10,38,39,40]. Studies on applications for these by-products have attracted great interest not only from an environmental point of view but also from an economic and human health perspective, as producing functional foods containing natural extracts from the olive plant Olea europaea could prove to be an excellent food industry strategy for improving human health, well-being and immune function [6].

Several phenolic compounds found in extra virgin olive oil (EVOO) may modulate inflammation by inhibiting pro-inflammatory enzymes or acting as antioxidants and the results obtained seem to confirm this hypothesis.

The commercial feed pellets made from OMWWs contained three main polyphenols: hydroxy-tyrosol, tyrosol and verbascoside [36], which have been proven to exert beneficial effects on liver cells in several studies [41,42,43].

Our hepatic tissue samples showed a decrease of *SIRT1* expression and significant differences were observed between the POL and CTR groups.

Sirtuins are DNA-dependent type-class III histone deacetylases (HDAC) whose catalytic activity is modulated by the metabolic state of the cells, i.e., by dynamic changes in NAD+ levels and the NAD+/NADH ratio. The requirement for NAD+ as co-substrate suggests that sirtuins may have evolved as sensors of energy and redox status in the cell. In general, these proteins are involved in lifespan and metabolism regulation by deacetylating the histones of some transcriptional regulator genes including NF-κB (Nuclear Factor kappa-light-chain enhancer of activated B cells). SIRTs affect multiple metabolic processes and pathways including circadian clocks, cell cycle, mitochondrial biogenesis and energy homeostasis [44,45]. In particular, the SIRT1 protein is known to protect cells against oxidative stress and DNA damage [46] and plays an important role in regulating energy homeostasis in response to nutrient availability [47]. One of the main metabolic effectors of SIRT1, the peroxisome proliferator-activated receptor gamma coactivator 1-alpha (PCG-1α), is activated by SIRT1-mediated deacetylation. Activated PGC-1α enhances hepatic gluconeogenesis and consequently promotes obesity prevention and averts metabolic dysfunction [48]. Recent studies have focused on the application of natural polyphenols for modulating epigenetic pathways through SIRT1 modulation and their effect on several chronic diseases [49].

The protective effects of tyrosol, a major component of OMWWs, are also due to the increase in SIRT1 protein expression, nuclear translocation and ERK1/2 phosphorylation which protect the cells against apoptosis [41].

In an in vitro model of hepatic steatosis, an olive leaf extract (OLE) containing verbascoside, another component of OMWWs, proved to protect against free fatty acid accumulation in hepatocytes [36,50]. This treatment ameliorated the lipid metabolism, led to a concurrent increase in FABP-4, SIRT-1 and HO-1 expression and significantly reduced the levels of the pro-inflammatory cytokines IL1β and TNFα.

The main plant phenolic compounds in olive oil such as hydroxy-tyrosol and oleuropein are known to activate molecular targets such as SIRT1 [42].

However, in our experiments the POL group showed decreased *SIRT1* expression compared to the CTR group. This result is in line with the findings of Chen et al. [51] who revealed that polyphenols can exert a similar caloric-restriction (CR) function by acting on SIRT1 expression, which is usually increased in many tissues, but appears decreased in the liver. Indeed, hepatic SIRT1 levels were reduced by CR which led to decreased hepatic fat synthesis and fat accumulation [51].

The other down-regulated genes in the POL group are *TNFA*, *AGER* and *BAX*. This modulation may be due to the ability of phenolic compounds to regulate the hepatic nuclear factor κB (NF-κB) pathway, which is a key transcription factor that enhances pro-inflammatory signaling. In fact, NF-κB regulates the expression of various effectors involved in inflammation (such as TNFA, AGER and BAX) and is activated by different stimuli including oxidative stress. Oxidative stress could link these genetic pathways in various ways. Indeed, Fki et al. [43] demonstrated that, in rats fed high-fat diets, the histological analysis of liver tissue revealed a significant increase in the expression of inflammatory genes (*COX-2*, *NFKB*, and *TNFA*) and apoptotic markers (a decrease in the expression of the Bcl-2 and an increase of the P53), while the oral administration of hydroxy-tyrosol-rich olive extracts attenuated liver inflammation and apoptosis.

By studying the hepatic tissue of monogastric species, Hu et al. [52] showed that some plant polyphenols, namely oleuropein and piceatannol, can modulate the mRNA expression levels of various oxidative stress-stimulated genes such as *TNFA*.

Advanced glycation end product (AGE) formation is a key pathophysiological event linked to the onset and progression of several non-infectious human diseases. AGEs mainly contribute to pathophysiology by forming cross-links and engaging the receptor for advanced glycation end-products (RAGE or AGER). One of the beneficial effects of polyphenols is their ability to limit the harmful consequences of advanced glycation (i.e., the disruption of cellular homeostasis. This anti-glycation activity occurs mainly by inhibiting ROS formation during glycation as well as by blocking AGE–RAGE interaction or signaling [53]. Most AGE-binding proteins are involved in AGE clearance mechanisms, mainly through endocytic uptake and degradation. However, one AGE-binding protein, known as the AGER receptor, can generate a robust pro-inflammatory response in numerous cell types once engaged. Ligation of AGER induces inflammatory gene expression profiles and leads to a positive feed-forward loop, in which inflammatory stimuli activate NF-κB and AGER expression, followed by a sustained NF-κB activation. For these reasons, in our experiments, the downregulation of *AGER* gene expression observed in the POL group compared to the CTR indicated the possible reduction of oxidative stress phenomena and inflammatory processes, which corroborates the results previously obtained in a study on rabbits carried out by Lin et al. [54].

Oxidative stress induces a number of cellular processes linked to inflammatory events as well as proliferation and apoptosis. Hepatocellular apoptosis is known to favor the pathogenesis of liver diseases, which is co-regulated by the Bcl-2 family, with Bax and Bcl-2 as key members, and the caspase family including caspase-3. The inhibition of oxidative stress and hepatic cell apoptosis effectively prevents liver damage characterized by inflammatory molecular events which are mostly NF-κB dependent [55]. Several studies conducted on experimental mouse models for induced-liver damage have demonstrated that tea polyphenols are able to reduce the up-regulation of pro-apoptotic genes [55,56] including *BAX* [57]. Furthermore piceatannol, a phenolic compound belonging to the stilbene family, has been proven to inhibit *BAX* gene expression in the livers of weaned piglets [58]. This is in line with our results, which showed a reduction in *BAX* gene expression in the POL rabbits’ liver tissues compared to the CTR group, thus suggesting that the antioxidant approach maybe an effective treatment against liver damage.

Consequently, the reduction in ROS inhibited NF-κB a pivotal mediator of inflammatory responses [59]. NF-κB is known to transcribe inflammatory markers such as interleukin 6 (IL6), interleukin 2 (IL2) and *TNFA* [52]. A decrease in these pro-inflammatory molecules, including *TNF*α, may be due to the polyphenol-induced inhibition of oxidative stress acting on NF-κB that triggered the down-regulation of *TNFA*, *AGER* and *BAX* expression as also observed in the POL group in our experimental protocol. This suggests that the antioxidant capacity of OMWW polyphenols may be due to their anti-inflammatory and anti-apoptotic activities like other botanical polyphenols found in tea, grape and curcumin [52,59,60,61,62]. Our results are also supported by the potential anti-inflammatory activity of an olive leaf extract in streptozotocin-induced diabetic rats’ livers, which inhibited the production of many inflammatory cytokines including TNFA [63].

A cited polyphenolic-induced indirect mechanism of NF-κB repression, with a consequent reduction in many pro-inflammatory genes, seems to occur via PPAR-ɣ activation [64]. However, in this study *PPARG* gene expression does not appear statistically significant between the POL and CTR groups (*p*-value = 0.161), even if many studies [66,67,68] have observed a polyphenol-induced regulation of lipogenesis and inflammation in the livers of different species. In our study the effect of OMWW polyphenols does not appear to involve the hepatic lipid metabolism.

PPAR-ɣ and PPAR-α are hepatic factors which modulate the activity of various target genes by regulating fatty acid metabolism. Several studies [65,66,67,68] have demonstrated that the polyphenol oleuropein, green tea polyphenols, the flavonoid quercetin and polyphenols from tea catechins induced PPARA expression in mouse livers, broiler chickens and hamsters.

However, in our study *PPARA* was lower in the POL group than in the CTR group. This may be due to the fact that lipid synthesis occurs at two major sites in animals: the liver and the adipose tissue, and the relative contribution of these sites to lipogenesis varies among species. Differences in the site of fatty acid synthesis and the pattern of lipid trafficking affect both overall animal lipid metabolism and the roles played by regulatory hormones and transcription factors. Moreover, in rabbit livers, transcription factors such as PPARα may prove to be less activated by anti-oxidant molecules such as polyphenols. However, this result may be due to dose-dependent effects of polyphenols.

Furthermore, no significant differences were observed between the *IL1B* and *COX2* inflammatory gene expression levels of the POL group and the CTR group. IL1B, an important inflammatory cytokine, showed a down regulation in the POL group and its lack of statistical significance may be linked to limited statistical power which would probably require a larger sample size for each experimental group.

The *COX2* gene encodes another important inflammatory molecule, the cyclooxygenase (COX)-2, which together with the COX1 isoenzyme, is involved in converting arachidonic acid into prostaglandins. Prostaglandins play essential roles in numerous biological processes, including the regulation of the immune system. Although the *COX1* gene is expressed in most tissues, *COX2* is strongly expressed under inflammatory conditions [69]. A recent study investigated whether the anti-obesity and anti-inflammatory properties of tea polyphenols are associated with the inhibition of COX2 and inducible nitric oxide synthase (NOS2) expression levels via the modulation of COX2 signaling pathways and a consequent inflammatory cytokine response in liver [47] and intestinal epithelial in dogs [70]. The possible mechanism underlying the decrease in COX2 protein expression is the activation of PPARG by polyphenols, which mediates the suppression of *COX2* and *NOS2* promoters [71]. However, in our experiments, the *PPARG* gene proved to be slightly downregulated, consequently the upregulation of *COX2* is expected.

In our experiments, *PPARG* expression was not modulated in the two experimental groups, which may have led to lack of modulation of *COX2*.

The data obtained in this study suggest that the phenols present at high concentrations in OMWW could be used as anti-oxidant supplements in commercial pelleted rabbit feed and transformed into a natural source of valuable antioxidants for enhancing both animal and human health. However, further research is required to confirm the beneficial effect of OMWW polyphenols.

Furthermore, extracts of *Olea europaea* polyphenols could be also regarded as natural anti-oxidant alternatives to synthetic additives for improving the preservation of meat and meat products, by preventing oxidation and microbial growth [72,73,74]. Interestingly, a recent study by Maranesi et al. [36] showed the effect of these dietary OMWW compounds on inflammatory and apoptotic processes through the modulation of COX2 and BAX expression in rabbit ovaries for the first time. Although there is still a gap in the knowledge of the effects of OMWW polyphenols on rabbits, recent studies have shown that different plant polyphenols (tea polyphenols, oleuropein, resveratrol, curcumin and piceatannol) can modulate the gene expression of various oxidative stress-stimulated genes including *TNFA*, *AGER* [54] and *BAX* [57,58] in the hepatic tissue of monogastric species [52,59,60,61,62]. In this study, the data obtained for *TNFA*, *AGER* and *BAX* genes suggest the beneficial role of dietary OMWW polyphenols in regulating hepatic oxidative stress and inflammation, defense system and apoptosis.

## 5. Conclusions

Recently researchers have shown great interest in the beneficial properties and lack of side effects of polyphenols obtained from natural sources. The large number and variety of bioactive molecules present in olive oil by-products have encouraged their usage as supplements to improve the performance, health and welfare of ruminants and mono-gastric animals in order to obtain functional foods for human consumption.

Our data suggest for the first time that OMWW dietary polyphenols are directly involved in many hepatic oxidative stress-dependent metabolic pathways of rabbits. In particular, our results indicate a possible involvement of OMWW dietary polyphenols in hepatic inflammatory and apoptotic events via the modulation of *TNFA*, *AGER* and *BAX* gene expressions. Although further studies are still required, these findings represent the first step towards protecting food-producing animals against the negative effects of oxidative stress by feeding them a diet supplemented with by-products from OMWW.

## Figures and Tables

**Table 1:** Primers used for gene quantification by qPCR and designed according to the sequences provided by Primer-BLAST Software.

Gene	Accession Number and Genomic Coordinates	Primer Forward	Primer Reverse
*ACTB*	NM_001101683.1 chrUn0180:126294-126816	CACCTTCTACAACGAGCTGC	TGTTGAACGTCTCGAACATGA
*RN18S*	Maranesi et al. [36]	CGATCAGATACCGTTCGTAGT	TTCCTTTAAGTTTCAGCTTTGC
*RN18S*	NR_033238.1 chrUn0416:102245-103489	CGTCTGCCCTATCAACTTTCG	AATGGGGTTCAACGGGTTAC
*AGER*	XM_002714315.3 chr12:20967567-20968080	CCACCCATCCCAACCGTG	GCTAGAGTCCCCAGGCCT
*BAX*	XM_008252361.2 chrUn0331:93,210-93,457	CCTTTTGCTTCAGGGTTCA	ATCCTCTGCAGCTCCATGTT
*COX2*	NC_001913.1 chrM:7201+7281	ACAATGGATGCTCAGGAGGT	TAGGGAGGGCAGCGCAATTA
*IL1B*	NM_001082201.1 chr2:97615039-97616359	GAATCTGTACCTGTCCTGCGT	TTGGGTAACGGTTGGGGTCT
*PPARA*	XM_002723354.3 chrUn0230:149032+156977	ATGAACAAGGTCAAAGCCCG	ATGAACAAGGTCAAAGCCCG
*PPARG*	NM_001082148.1 chr9:11539648-11541491	CCTTTCACCACCGTGGACTT	GGGGATGCAGGTTCCACTTT
*SIRT1*	XM_017348747.1 chr18:18711449-18715261	TGCAAGCTCTAGTGACTGGA	TGTTCGAGGATCTGTGCCAA
*TNFA*	NM_001082263.1 chr12:20399776+20400811	ACTTCAGGGTGATCGGCC	CCTCCACTTGCGGGTTTG

## Data Availability

The data presented in this study are available on request from the corresponding authors.
